# Irrigated urban trees exhibit greater functional trait plasticity compared to natural stands

**DOI:** 10.1098/rsbl.2022.0448

**Published:** 2023-01-04

**Authors:** Peter C. Ibsen, Louis S. Santiago, Sheri A. Shiflett, Mark Chandler, G. Darrel Jenerette

**Affiliations:** ^1^ Department of Botany and Plant Sciences, University of California Riverside, Riverside, CA 92521, USA; ^2^ Geosciences and Environmental Change Science Center, United States Geological Survey, Denver, CO 80225, USA; ^3^ Department of Environmental Sciences, University of North Carolina Wilmington, Wilmington, NC 28403, USA; ^4^ Earthwatch Institute, Boston, MA 02143, USA

**Keywords:** aridity, community science, carbon gain, novel ecosystem, urban trees, water use

## Abstract

Urbanization creates novel ecosystems comprised of species assemblages and environments with no natural analogue. Moreover, irrigation can alter plant function compared to non-irrigated systems. However, the capacity of irrigation to alter functional trait patterns across multiple species is unknown but may be important for the dynamics of urban ecosystems. We evaluated the hypothesis that urban irrigation influences plasticity in functional traits by measuring carbon-gain and water-use traits of 30 tree species planted in Southern California, USA spanning a coastal-to-desert gradient. Tree species respond to irrigation through increasing the carbon-gain trait relationship of leaf nitrogen per specific leaf area compared to their native habitat. Moreover, most species shift to a water-use strategy of greater water loss through stomata when planted in irrigated desert-like environments compared to coastal environments, implying that irrigated species capitalize on increased water availability to cool their leaves in extreme heat and high evaporative demand conditions. Therefore, irrigated urban environments increase the plasticity of trait responses compared to native ecosystems, allowing for novel response to climatic variation. Our results indicate that trees grown in water-resource-rich urban ecosystems can alter their functional traits plasticity beyond those measured in native ecosystems, which can lead to plant trait dynamics with no natural analogue.

## 1. Introduction

Urbanization is a recognized cause of novel ecosystems, assemblages of species living in environments with no natural analogue [1,2]. Novel urban tree communities result from including species from globally distributed biogeographic provinces [3], and trees encountering factors that differ from natural stands, from soil characteristics to variable microclimates [4,5]. Another cause of novel tree communities is greater resource availability, notably irrigation, in the growing environment [6]. The effect of urban irrigation on tree functioning can alter mortality, growth and phenology [7,8], which may drive changes in important plant traits. The potential for novel functional trait distributions as an additional effect of irrigated urbanization and novel ecosystem in general is unknown.

Carbon gain and water use reflect trait variation axes linked to tree physiological functioning and may change in response to urban irrigation. Trait suites associated with carbon gain and water use reflect the trade-offs between high resource use and faster growth on one end of the spectrum, and a reduced resource use and more conservative growth on the other [9]. The leaf economic spectrum (LES) describes the carbon-gain axis [10] and is exemplified by the positive relationship between specific leaf area (SLA) and leaf nitrogen concentration (%N) [11]. SLA and %N indicate allocation to maximum carbon-gain potential, which is associated with LES position, varying from fast to slow return on carbon investment to photosynthetic structures [10]. An analogous spectrum considers leaf water-use strategies [12], where conservative strategies enable preservation of internal water resources in the presence of soil or atmospheric drought. This trade-off is demonstrated in the relationship between stomata guard cell length (GCL) and stomata density, where longer length and low-density values are associated with rapid water transport and loss [13]. Physiological coupling of carbon gain and water use can be represented by negative relationships between wood density (WD) and minimum daily leaf water potential, where greater carbon gain is associated with increasing water transport capacity in the xylem [14]. However, how these trait distributions and their coupling are expressed and vary in irrigated urban trees is uncertain [15].

Irrigated urban trees may functionally differ from tree species planted within natural stands in carbon-gain and water-use traits. Urban irrigation could favour greater carbon-gain and water-use traits, with effects increasing in arid environments where enhanced evaporative cooling would be beneficial [16]. Moreover, irrigation may decouple carbon gain and water use, where instead of acting as a trade-off, these processes run independently of each other [17]. This decoupling provides opportunities for plants to plastically respond to climatic changes. During extreme heat, carbon-gain rates may become decoupled from transpiration rates through access to soil moisture. [18,19]. The slowing of photosynthetic processes during extreme heat, while increasing leaf transpiration is likely facilitated by available subsurface water and potentially leads to more liberal water-use strategies which may protect leaves from heat damage caused by more frequent future heatwaves [20,21]. Preventing soil drought through irrigation in arid environments can contribute to this decoupling, creating a disconnect between atmospheric water demand and water availability [22,23].

To identify consequences of urban high water resources on trees and the potential for novel trait distributions, we ask how does an irrigated urban environment affect tree carbon-gain and water-use strategies? To answer this question, we assessed trait values throughout the Los Angeles, USA megacity. We use Los Angeles as a model urban ecosystem spanning a coastal Mediterranean to arid desert climate gradient with regular irrigation with extensive tree biodiversity [3]. We hypothesized that trees respond to urban irrigation by simultaneously increasing the capacity for carbon gain and water transport. We tested the prediction that high water resources found in irrigated urban systems alter trait relationships for both carbon-gain and water-use traits compared to the same species in their native habitats. We also tested the prediction that effects of irrigation would depend on climate such that increased aridity leads to elevated carbon-gain traits and more liberal water-use strategies. Our study aims to resolve uncertainties of how key trait values and relationships respond to urban irrigation and highlight an under-recognized component of novel ecosystems.

## 2. Methods

### (a) Study system

The Los Angeles Megacity comprises over 17.5 million residents and is highly urbanized from the coast to the Coachella Valley desert. Atmospheric aridity, measured as the difference between mean atmospheric-saturation water vapour pressure and actual water vapour pressure in the air (vapour–pressure deficit; VPD), and maximum summer temperatures range from approximately 1.4 kPa and approximately 24.5°C on the coast, to approximately 6.1 kPa and approximately 41.0°C in the desert [24]. We use mean VPD as a primary climate variable, as temperature and VPD are tightly correlated across this region [25].

### (b) Data collection

To assess trait distributions, we selected 30 tree species (electronic supplementary material, appendix S2; table S1) representing 11 biomes of origin, and included eight of the 15 most common Southern California street trees [3]. We located sample individuals via partnership with trained local community scientists. Community scientists identified potential individuals, recorded GPS location and provided a qualitative evaluation of tree condition and surrounding environment. We focused on healthy irrigated specimens by only sampling community scientists identified healthy trees with at least 65% irrigated area surrounding a 10 m radius around the base of the tree.

We quantified the ‘carbon-gain’ train suite by being comprised of SLA, %N and leaf laminar thickness (LT) and the ‘water-use’ trait suite being comprised of GCL, stomatal density (SD) and WD. We calculated water-use strategy shifts as the coastal-to-desert variation between leaf water potential at pre-dawn (*Ψ*_PD_), midday (*Ψ*_MD_) and their daily difference (Δ*Ψ*_L_). Values of *Ψ*_PD_ approximate night-time equilibrium with soil water potential, and values of *Ψ*_MD_ and Δ*Ψ* reflect daytime plant water status, with more negative values indicating a liberal water-use strategy [26]. Sampling procedures are included in electronic supplementary material, appendix S1, and trait data are available from a Dryad dataset [27].

To compare urban carbon-gain and water-use strategies to trees in natural habitats, we obtained LES trait values (SLA and %N), and water-use traits (*Ψ*_PD_ and WD) for each species from their native habitats with the TRY database and primary sources and compared their linear regressions slopes [28–30]. Statistical analyses were completed in RStudio version 1.4.1106 [31].

## 3. Results

**Figure 1.**
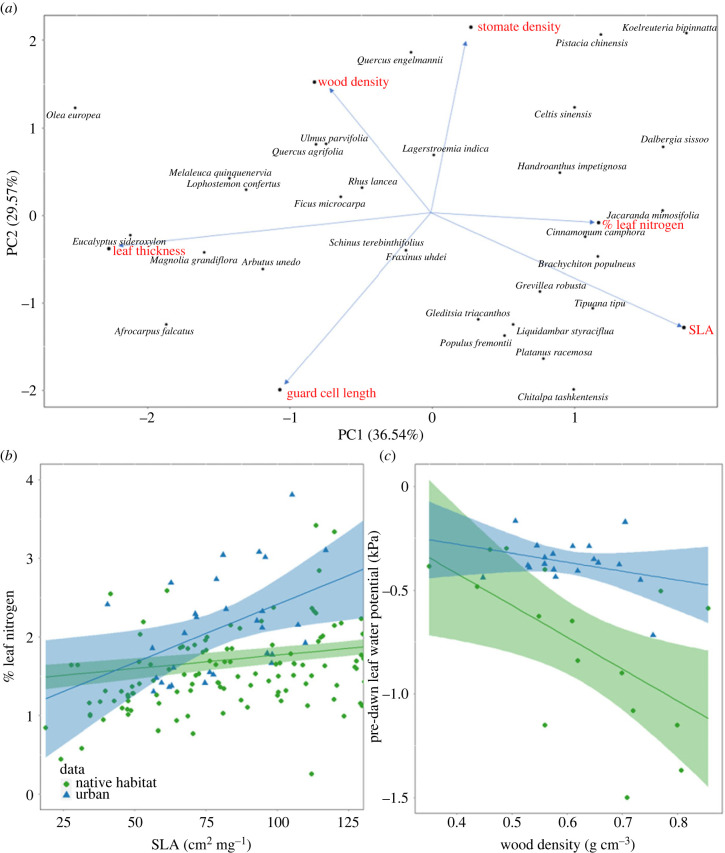
(*a*) Principal components analysis of trait variation across all study species. Percentage next to axis label indicates the variation in trait values determined by that axis. (*b*,*c*) Trait coordination relationships for (*b*) two carbon-gain traits, SLA and per cent leaf N by mass (%N) and two (*c*) water-use traits are represented as the relationship between WD and pre-dawn leaf water potential. Blue solid lines represent the linear regression and 95% confidence interval of urban sampled data when linear regression is significant (*α* < 0.05). Green solid lines represent the linear regression and 95% confidence interval of data species representing the trait spectra in native habitats (*α* < 0.05). Slopes of urban and native habitats are significantly different for (*b*) and (*c*) ((*b*): *p* = 0.002, (*c*) *p* < 0.001).

We found the two primary axes of the principal component analysis ordination comprised 65% of plant trait variation (axis 1 = 36.5% variation, axis 2 = 29.6% variation) (figure 1*a*). PC axis 1 was associated with carbon-gain traits (SLA – loading value: 0.58, %N – loading value: 0.39, LT – loading value: –0.64). PC axis 2 was mostly associated with water-use traits (GCL – loading value: −0.42, SD – loading value: 0.69, WD – loading value: 0.53). Carbon-gain traits were significantly correlated with each other (SLA, %N; *r* = 0.31, SLA, LT; *r* = −0.59, %N, LT; *r* = −0.18). Some water-use traits were significantly correlated (SD, GCL; *r* = −0.3, SD, WD; *r* = 0.25), although no significant correlation was observed between GCL and WD (table 1).

**Table 1.** Correlation matrix of interactions across traits and aridity (VPD) of all sampled individuals. Only significant (*p* < 0.05) correlation coefficients are bolded; non-significant coefficients are in italics.

	SLA	leaf nitrogen	leaf thickness	stomate density	GCL	WD
VPD	**0.25**	*0.12*	*−0.09*	*0.05*	*0.04*	*0.01*
SLA		**0.31**	**−0.59**	*−0.05*	*−0.1*	**−0.33**
leaf nitrogen			**−0.18**	*0*	**−0.12**	*−0.07*
leaf thickness				*−0.07*	**0.19**	**0.21**
stomate density					**−0.3**	**0.25**
GCL						***−**0.05*

Urban trees differed from natural stands in both carbon-gain and water-use trait values showing greater leaf N per unit SLA (figure 1*b*; difference between slopes: *p* = 0.002; urban: slope = 0.015, Adj *R*^2^ = 0.167, *p* = 0.014; native habitat: slope = 0.003, Adj *R*^2^ = 0.157, *p* < 0.001). Urban trees displayed greater pre-dawn leaf water potential per unit WD (figure 1*c*) compared to the natural stands. Urban trees exhibited no relationship between *Ψ*_PD_ and WD, while native counterparts displayed coupling of water-use and carbon-gain traits through a negative correlation between these traits (figure 1*c*; difference between slopes: *p* < 0.001; urban: *p* = 0.210; native: *p* = 0.016).

### (a) Interactions among species, traits and climate

Carbon-gain traits were positively correlated with aridity across all sampled individuals (VPD, SLA; *r* = 0.22, VPD, %N; *r* = 0.13) (table 1). Within individual species, we observed more correlations between traits and the local climate, nevertheless, there was no singular trait approximately VPD relation which was significant for all species. Similarly, no species displayed significant correlations between all traits approximately VPD. Twenty species exhibited significant correlations among traits and climate, representing 76% of all sampled species. All within-species significant correlations between carbon-gain traits and VPD were positive, whereas correlations of other traits as a function of VPD varied between positive and negative depending on species and trait (table 2).

**Table 2.** Pearson correlation coefficients between functional traits and VPD, restricted to intraspecific variation within each study species. Significant (*p* < 0.05) coefficients are in bold; non-significant are italicized.

species	SLA	functional trait		stomate density	GCL	WD
		leaf nitrogen	leaf thickness			
*Afrocarpus falcatus*	*−0.31*	*−0.16*	*0.068*	**0.71**	**0.93**	**0.44**
*Arbutus unedo*	*0.6*	*0.71*	*−0.14*	*−0.026*	*−0.39*	*0.47*
*Brachychiton populneus*	*0.77*	*0.23*	*−0.95*	*0.42*	*−0.47*	*0.5*
*Celtis sinensis*	*0.49*	*−0.95*	*0.12*	*0.24*	*−0.51*	*0.5*
*Chitalpa tashkentensis*	*−0.72*	−1	***1.42***	*−0.184*	**1.5**	*0.58*
*Cinnamomum camphora*	**0.65**	*0.5*	*−0.38*	*0.42*	**−0.62**	*−0.73*
*Eucalyptus sideroxylon*	*0.55*	*−0.0036*	*0.43*	*0.12*	*−0.4*	*−0.092*
*Ficus microcarpa*	**0.7**	*−0.16*	*−0.36*	*0.7*	**−0.66**	*−0.38*
*Fraxinus uhdei*	*−0.29*	*0.22*	*0.32*	*0.52*	**0.73**	*0.4*
*Gleditsia triacanthos*	*0.2*	*0.19*	*0.31*	*0.54*	*0.34*	*0.67*
*Grevillea robusta*	**−0.2**	*0.067*	**−1**	*0.36*	*0.53*	*−0.97*
*Handroanthus impetignosa*	*0.28*	*−0.54*	*0.061*	*0.25*	*−0.41*	*0.63*
*Jacaranda mimosifolia*	*−0.093*	*0.099*	*−0.47*	*−0.37*	*0.21*	*−0.5*
*Koelreuteria bipinnata*	*−0.57*	*−0.59*	*0.56*	*−0.28*	*−0.12*	*0.48*
*Lagerstroemia indica*	**0.68**	*0.14*	*−0.33*	*−0.24*	*0.48*	*−0.093*
*Liquidambar stryaciflua*	*0.57*	*0.26*	*−0.15*	*−0.15*	*−0.54*	*−0.33*
*Lophostemon confertus*	*−0.35*	*−0.27*	*0.046*	*−0.65*	**0.85**	*−0.033*
*Magnolia grandiflora*	*0.052*	*0.36*	*0.24*	**−0.62**	*0.13*	*−0.061*
*Melaleuca quinquenervia*	*0.67*	*−0.44*	**−0.68**	*0.32*	*−0.32*	*−0.46*
*Olea europaea*	*0.35*	*−0.49*	*0.11*	*0.47*	*0.049*	*−0.19*
*Pistacia chinensis*	*0.43*	**0.9**	*−0.082*	*0.31*	**−0.81**	*−0.64*
*Platanus racemosa*	**0.54**	*0.21*	*0.021*	*0.36*	*0.39*	**0.62**
*Populus freemontii*	*−0.18*	*0.024*	*0.59*	**0.88**	*−0.69*	*0.38*
*Quercus agrifolia*	*−0.25*	**0.57**	*−0.31*	*−0.13*	*0.3*	**−0.57**
*Quercus engelmanii*	*0.58*	*0.18*	*−0.46*	*0.001*	*0.68*	**−0.81**
*Rhus lancea*	*−0.52*	*0.36*	*0.13*	*−0.012*	*0.34*	*0.53*
*Schinus terebinthefolius*	**0.66**	*−0.035*	**−0.69**	*0.73*	*−0.64*	*0.035*
*Tipuana tipu*	*0.28*	*−0.33*	*−0.1*	*−0.21*	*−0.38*	*−0.099*
*Ulmus parvifolia*	*0.031*	*−0.6*	*−0.19*	*0.61*	*−0.54*	**0.77**

**Figure 2.**
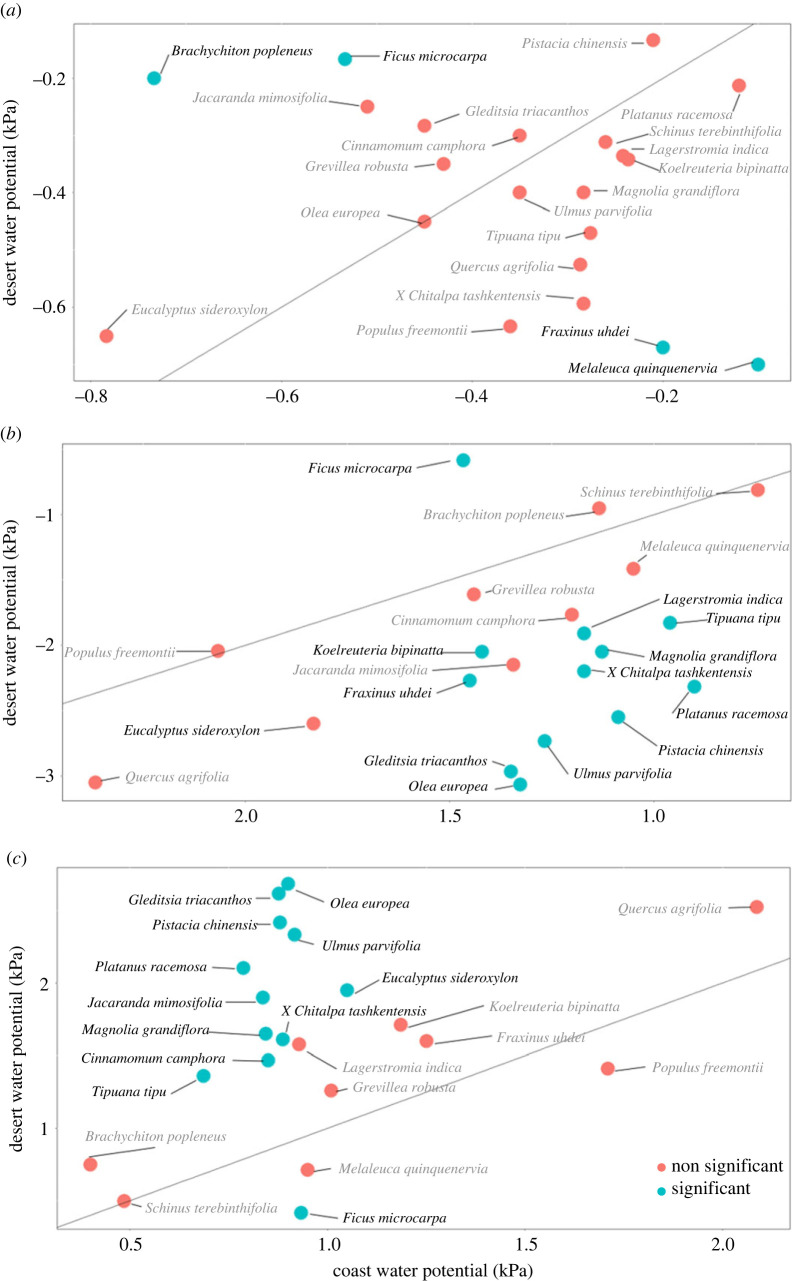
Comparison of water-use in urban tree species planted near the coast versus the desert. Water-use change is described by: (*a*) difference between species pre-dawn leaf water potential from coast to desert, (*b*) difference between species midday leaf water potential from coast to desert and (*c*) delta leaf water potential (midday–pre-dawn) from coast to desert. The 1 : 1 line indicated no difference between coast or desert water use. The water-use parameter of species with red points and greyed names was not significantly different between locations.

Across the climate gradient, water-use strategy within urban species varied from coastal to desert regions. Out of 21 species, four exhibited differences in coastal and desert *Ψ*_PD,_ 13 for *Ψ*_MD_ and 11 species shifted mean *ΔΨ*_L_ between urban coast and desert (*p* < 0.05). For *Ψ*_MD_ and *ΔΨ*_L_, all species that shifted water-use strategies tended towards more liberal water use, except *Ficus microcarpa* (figure 2). Six species increased Δ*Ψ*_L_ in desert environments by more than a factor of two.

## 4. Discussion

In greater Los Angeles, urbanization with irrigation leads to novel functional trait relationships. Irrigated urban trees generally exhibited greater carbon gain relative to trees in their native habitats. Our results show irrigated urban trees pushing the bounds of the LES capacity for carbon gain while reducing constraints to water losses. The combination of increased carbon trait relationships and weakened water-use relationships results in a decoupling of carbon-gain and water-use strategies in urban trees. With the addition of irrigation, urban trees have an unexpected capacity to present novel water-use and carbon responses to extreme heat and aridity, previously unmeasured in multi-species studies. We interpret these results as evidence that functional traits of irrigated urban trees diverge from relationships observed in native habitats to accelerate carbon gain and increase water loss, while increasing aridity magnifies these effects. Irrigation-induced trait changes and their sensitivity to aridity contribute a functional component to novel urban ecosystems beyond changes in environments and community assemblages.

While the urban tree traits were distributed into two axes associated with carbon gain and water use, carbon-gain and water-use strategies deviated from their respective relationships in natural stands in contrasting ways. We interpret this as carbon trait relationships are amplified in irrigated urban settings and water-use trait relationships are weakened compared to native conditions. Within native habitats, resource availability can determine ecological trade-offs in functional trait suites [32]. The heightened ratio of N : SLA of resource-saturated trees compared to natural habitats is consistent with patterns of accelerated growth found in other urban forests, creating a potential trade-off between increased carbon sequestration at the cost of rapid mortality [33]. Carbon trait responses occur in the context of reduced sensitivity of leaf water-use traits to the constraints of xylem architecture, providing longer periods of carbon acquisition at the leaf level. Our finding is consistent with studies showing that increased soil moisture results in trees lessening stomatal control [34], allowing for a ‘faster’ carbon gain in environments of higher VPD. While such carbon and water-use trait decoupling can also occur in tropical and sub-tropical trees growing in natural stands where soil moisture is not limiting [19], as the urban trees of the Los Angeles area represent species native to biomes across the planet, our results suggest global trait flexibility of diverse species planted in semi-arid urban environments when irrigation is applied.

The decoupling between carbon-gain and water-use strategies may facilitate increased functioning in environments of extreme heat or atmospheric aridity than previously expected. Correspondingly, the effect of urban irrigation on tree carbon-gain and water-use traits also varied in response to climate. Throughout our study, urban tree carbon-gain traits were positively correlated with aridity, which is in contrast with the expected decrease in tree growth in response to increases in VPD [22]. Concurrently, water-use strategy shifted to a more liberal strategy in desert environments (figure 2), implying that trees from diverse habitats can plastically alter water-use strategies, meeting atmospheric demands when adequate soil moisture is available. The ability to use available water therefore facilitates an increase in carbon gain and explains the contrasting effects of long-term climate and irrigation and points to another aspect of novel ecosystem trait distributions. Shifting water-use responses mirror mechanistic predictions by Wolf *et al*. [35] where under well-watered conditions, plants that favour water-use-efficiency strategies, and plants that favour carbon-maximizing stomatal strategies will act similarly. These results occurred even for tree species that never naturally experience the extreme heat and arid conditions found in the desert. The combination of irrigation and extreme climates in arid urban cities shows how human facilitation and trait flexibilities create novel functional compositions, as increased functional plasticity opens up the arid urban species pool to greater diversity [36,37].

Using a metropolitan region spanning a dramatic climate gradient provides a unique ‘common garden’ to broadly evaluate how irrigation influences tree functional traits and to examine fundamental ecological relationships in urban environments [38]. While urban environments have been considered laboratories to study species composition due to their global diversity of tree species [39] and effects of global change [40,41], using this laboratory we found that urban irrigation is associated with distinct functional trait relationships. These findings further highlight the importance of the distinct effects of atmospheric drought separately from soil drought. The unexpected responses of trees to increased water resources are an important constraint when evaluating future functioning of urban forests. We focused on irrigation as a cause of the high-water resource availability which can influence tree function; however, the urban environment also contains other potential sources of high-resource availability, including CO_2_, nitrogen and heat, all which can influence function [42–44]. Within cities, policy has called for increased planting of ‘drought-tolerant’ species [45]. Yet, if cities do not change irrigation practices, our results suggest ‘drought-tolerant’ species may ultimately increase capacity for water use. Our study demonstrates in an urban ecosystem, as an example of a novel ecosystem, trees exhibit unexpected flexibility in ecological strategies.

## Data Availability

Raw data used for analysis are available from the Dryad Digital Repository: https://doi.org/10.6086/D1N96B [27]. Data include a .csv file of all leaf and stem trait data collected and analysed in the study for each sampled tree (RT_all.csv), a .csv file of leaf water potential measurements for all trees used in the study (RT_water_potential.csv) and a README file with metadata describing column names, descriptions and units. Supplementary material is available online [46].
